# In-hospital mortality from healthcare-associated infection by multidrug-resistant Pseudomonas aeruginosa: a competing risks analysis of a 4-year propensity-matched cohort study in southern China

**DOI:** 10.3205/dgkh000597

**Published:** 2025-11-21

**Authors:** Mouqing Zhou, Evangelos I. Kritsotakis, Baohua Xu, Zhusheng Guo, Yongfeng Zeng, Bin Zhou, Ralph Brinks, Jiancong Wang

**Affiliations:** 1 Department of Infection Control, DongGuan SongShan Lake Tungwah Hospital, DongGuan, Guangdong Province, China; 2 Laboratory of Biostatistics, Division of Social Medicine, School of Medicine, University of Crete, Heraklion, Greece; 3 Department of Science Research, and Education, DongGuan Tungwah Hospital, DongGuan, Guangdong Province, China; 4 Department of Microbiology, DongGuan Tungwah Hospital, DongGuan, Guangdong Province, China; 5 Department of Infection Control, DongGuan Tungwah Hospital, DongGuan, Guangdong Province, China; 6 Chair of Model-based Environmental Exposure Science, Faculty of Medicine, University of Augsburg, Augsburg, Germany; 7 Chair for Medical Biometry and Epidemiology, University of Witten/Herdecke, Witten, Germany

**Keywords:** healthcare-associated infections, multidrug-resistant Pseudomonas aeruginosa, in-hospital mortality, discharge alive, competing risk analysis, antimicrobial stewardship, incidence density, China, Dongguan

## Abstract

**Background:**

Healthcare-associated infections (HAIs) caused by multidrug-resistant *Pseudomonas* *aeruginosa* (MDRPa) pose enormous challenges in healthcare. We examined the incidence and relative mortality rates of patients with MDRPa HAI compared to non-MDRPa HAI in southern China.

**Methods:**

A hospital-wide longitudinal cohort study was conducted using prospectively collected surveillance data from 2018 to 2021. Poisson regression was applied to estimate incidence rate ratios (IRRs). Propensity-score matching and competing risks regression analysis (Fine-Gray model) were employed to estimate subdistribution hazard ratios (sHRs) for in-hospital mortality comparing MDRPa to non-MDRPa infections.

**Results:**

Among 562 patients studied (mean age 58 years, 74% male, in-hospital mortality 13.7%), 278 (49%) had an MDRPa HAI and 284 (51%) a non-MDRPa HAI. The incidence rate of MDRPa HAIs increased over time (mean monthly IRR: 1.016, 95% CI: 1.007–1.024). No significant difference in 14-day in-hospital mortality between MDRPa and non-MDRPa HAIs were detected in the propensity-matched doubly-robust analysis (adjusted sHR: 1.07, 95% CI: 0.52–2.19). However, MDRPa HAI was associated with a lower probability of 14-day discharge alive (adjusted sHR: 0.44, 95% CI: 0.31–0.63), resulting in longer hospital stays.

**Conclusions:**

The study provided real-world evidence of the clinical burden of MDRPa HAIs in China, highlighting their rising incidence and direct effect on prolonging hospitalisation. The findings underscore the need for antimicrobial stewardship interventions to ensure timely de-escalation and optimised antibiotic therapy.

## Zusammenfassung

**Hintergrund:**

Gesundheitseinrichtungen stehen vor großen Herausforderungen durch gesundheitssystem-assoziierte Infektionen (auf Englisch: Healthcare-associated infections (HAIs)), die durch multiresistente *Pseudomonas* *aeruginosa* (MDRPa) verursacht werden. Diese Studie untersuchte die Inzidenz und die relative Sterblichkeit von Patient:innen mit MDRPa-HAI im Vergleich zu nicht-MDRPa-HAI in Südchina.

**Methoden:**

Es wurde eine krankenhausweite, longitudinale Kohortenstudie mit prospektiv erhobenen Überwachungsdaten aus den Jahren 2018 bis 2021 durchgeführt. Mittels Poisson-Regressionen wurden Inzidenzratenverhältnisse (IRRs) geschätzt. Um die Subverteilungs-Hazard-Ratios (sHRs) der intrahospitalen Sterblichkeit im Vergleich zwischen MDRPa- und nicht-MDRPa-Infektionen zu ermitteln, kamen das Propensity-Score-Matching sowie eine Competing-Risks-Regression (Fine-Gray-Modell) zum Einsatz.

**Ergebnisse:**

Unter den 562 untersuchten Patient:innen (Durchschnittsalter 58 Jahre, 74% männlich, intrahospitale Sterblichkeit 13,7%) hatten 278 (49%) eine MDRPa-HAI und 284 (51%) eine nicht-MDRPa-HAI. Die Inzidenzrate der MDRPa-HAIs stieg im Zeitverlauf an (durchschnittliches monatliches IRR: 1,016; 95%-KI: 1,007–1,024). In der doppelt robusten Analyse nach Propensity-Matching wurde kein signifikanter Unterschied in der 14-Tage-Sterblichkeit im Krankenhaus zwischen MDRPa- und nicht-MDRPa-HAIs festgestellt (adjustiertes sHR: 1,07; 95%-KI: 0,52–2,19). Allerdings war eine MDRPa-HAI mit einer geringeren Wahrscheinlichkeit verbunden, innerhalb von 14 Tagen lebend entlassen zu werden (adjustiertes sHR: 0,44; 95%-KI: 0,31–0,63), was zu längeren Krankenhausaufenthalten führte.

**Schlussfolgerungen:**

Die Studie liefert praxisnahe Evidenz für die klinische Belastung durch MDRPa-HAIs in China und hebt deren zunehmende Inzidenz sowie ihren direkten Einfluss auf verlängerte Krankenhausaufenthalte hervor. Die Ergebnisse unterstreichen die Notwendigkeit von Maßnahmen zu einer bedachtsamen Antibiotikaverwendung, um eine rechtzeitige Deeskalation und optimierte antimikrobielle Therapie sicherzustellen.

## Introduction

Treating healthcare-associated infections (HAIs) caused by multidrug-resistant *Pseudomonas (P.) aeruginosa* (MDRPa) is a considerable challenge for clinicians and the healthcare system [1]. In 2024, the World Health Organisation (WHO) classified MDRPa as a “high priority” pathogen on its Bacterial Priority Pathogens List, given its high transmissibility, elevated case fatality rates, and significant economic burden on healthcare facilities [1], [2], [3], [4]. Recent global data indicate an increasing trend in the incidence of MDRPa HAIs [5]. This rise has been largely attributed to the disruption of routine infection-control practices during the COVID-19 pandemic and the challenges in effectively implementing antimicrobial stewardship programs [1], [5], [6]. Furthermore, the lack of new antibiotics active against multidrug-resistant Gram-negative bacteria has resulted in limited treatment options, often leaving last-resort antibiotics as the only viable therapies [7]. Additionally, the growing elderly population requiring intensive care unit (ICU) admission, combined with an increase in comorbidities such as cancer, organ transplants, and immunocompromised conditions, has heightened the risk of acquiring MDRPa during hospitalisation [1].

As outlined in the susceptible-infection counterfactual framework by Karakonstantis et al. [7], a methodologically rigorous assessment of the impact of MDRPa HAIs on in-hospital mortality is essential. Competing risks survival analysis is becoming increasingly common in antimicrobial resistance research because traditional survival analysis may overestimate infection-related risks by ignoring competing outcomes, such as discharge alive [8], [9], [10]. However, despite its advantages for hazard estimation, only a few studies to date have applied this method to in-hospital mortality rates for MDRPa HAIs. For example, von Cube et al. [11] used multivariable competing risks analysis to assess overall ICU mortality by comparing ventilator-associated pneumonia (VAP) caused by *P. aeruginosa* with VAP not caused by *P. aeruginosa*. Similarly, Kritsotakis et al. [12] used multivariable competing risks analysis to examine in-hospital mortality at 14 and 30 days following the onset of ESKAPEE-associated bacteraemia – including *P. aeruginosa* – comparing MDR to non-MDR infections. However, to our knowledge, no studies have so far simultaneously applied both propensity score matching and multivariable competing risks analysis for MDRPa HAIs. Our study therefore aims to fill this methodological gap and to offer a clearer understanding of the mortality implications specific to MDRPa HAIs in order to inform clinical decision-making related to infection control using robust analytical methods. 

Against this background, the objectives of the present study were to

1. quantify the trend of incidence of MDRPa and non-MDRPa HAIs over time and
2. assess the excess in-hospital mortality from MDRPa HAIs relative to non-MDRPa HAIs in hospitalised patients in southern China.

## Methods

### Study design

A longitudinal cohort study was conducted at a 2,430-bed tertiary care, university-affiliated hospital in Dongguan City, located in the Guangdong-Hong Kong-Macao Greater Bay Area, in China. The cohort comprised hospitalised patients, regardless of age or department of admission, who had a confirmed HAI caused by *P. aeruginosa* as a monomicrobial infection, and were admitted on or after January 1, 2018, and discharged before December 31, 2021. Patients were included once in the study, and only their first episode of infection was considered [13].

### Outcome endpoints

The outcome endpoints of interest were the incidence rate of HAI due to *P. aeruginosa* and the all-cause in-hospital mortality rate, stratified by MDR status. Inpatient death within 14 days of the infection onset was considered the primary event of interest, as this is likely to be directly related to the infection. Additionally, 30-day and total in-hospital mortality rates were examined to evaluate the potential effect on delayed fatalities.

### Data collection

Prospectively collected data were extracted from the Dongguan Nosocomial Infection Surveillance System [14], [15]. Patient-related data (age, sex, diagnosis and department of admission, comorbidities), infection-related data (date of onset and site of infection), receipt of empiric therapy, and patient outcome (in-hospital death or discharge alive) were retrieved. 

### Definitions

A HAI was defined as an infection that was not present at the time of hospital admission, and was either acquired at least 48 hours after admission, or occurred within 30 days after surgery or other clinical intervention at another healthcare facility [16]. HAIs were confirmed clinically and microbiologically by the hospital’s clinicians using the diagnostic criteria for nosocomial infection published by the Ministry of Health of the People’s Republic of China [16], [17]. These definitions categorise HAI according to the organ/tissue system affected. The major infection sites considered for analysis were bloodstream infection, lower respiratory tract infection (including pneumonia), VAP, urinary tract infection and catheter-associated infection. All other types of infection were categorised as “other”. 

MDRPa status was declared for isolates non-susceptible to at least one antimicrobial agent in three or more antimicrobial groups, whereas non-MDRPa was declared when the isolates were non-susceptible to no more than two antimicrobial categories [13]. Antimicrobial susceptibility was assessed using the US National Clinical and Laboratory Standards Institute guidelines [15].

### Statistical analysis

Temporal changes in MDRPa and non-MDRPa HAI incidence were examined with a Poisson regression model to describe the variation of monthly incidence rates (number of infections per 1,000 hospitalisation days), for each major site of infection, with the time (in months) elapsed since the start of the study. The monthly series of hospitalisation days was used as an offset variable (log transformed) to account for the size of the hospital population and the length of hospital stay. This approach allowed the estimation of the mean monthly incidence rate ratio (IRR) and its 95% confidence interval (CI). IRR>1 indicates an upward trend and IRR<1 a downward trend of infection incidence over time. The mean monthly percentage change in infection incidence was calculated as (IRR–1)x100%. 

A propensity score-matched sample was constructed to minimise bias when comparing in-hospital mortality rates between patients with MDRPa HAI and those with non-MDRPa HAI. Propensity scores were estimated by logistic regression, accounting for age, sex, department at hospital admission, site of infection, diabetes, immunocompromised status, and COVID-19 period. Matching 1:1 was achieved by applying a nearest-neighbour method with a calliper width of 0.2 times the standard deviation of the logit of the propensity score using the MatchIt R package [18], [19]. Standardised mean differences less than 10% were considered to indicate an acceptable balance of covariates between groups [18], [19].

Cumulative probabilities of a patient dying in the hospital before any given day were calculated using the Aalen-Johansen method [20]. In this analysis, being discharged alive was treated as a competing event to in-hospital death [21]. The results were illustrated by cumulative incidence function (CIF) plots produced with the cuminc function of the cmprsk package in R. When comparing mortality and discharge-alive rates between MDRPa and non-MDRPa HAIs, the effect sizes were expressed as subdistribution hazard ratios (sHRs) with 95% confidence intervals from the Fine–Gray model, through the FGR function of the risk Regression R package [22]. The sHRs described the relative effect of MDR status and other covariates on the subdistribution hazard functions for (thereby, the probabilities of) in-hospital death and discharge-alive [22]. A low sHR for discharge-alive (<1) indicates a reduced daily discharge rate, leading to prolonged hospitalisation. 

The results from bivariable and multivariable Fine–Gray models were shown for both the original unmatched cohort and the propensity-matched sample. The multivariable regression analysis of the matched data, incorporating the variables used in the propensity-score model, can be regarded as a doubly robust adjustment [18]. Nine baseline covariates were included: age over 65 years, sex, admission diagnosis, department of admission, site of infection, receipt of empiric therapy, presence of diabetes, immunocompromised status, and year of infection occurrence. Multicollinearity of the covariates was ruled out by examining variance inflation factors (see Supplementary Table S1 in Attachment 1). 

For all survival analyses, time zero was defined as the time of infection onset. For the 14-day, 30-day and overall hospitalisation outcomes, event-free time was administratively censored at 14, 30 and 120 days, respectively, for patients who remained hospitalised for longer periods. There were no missing data for any study variable. Statistical significance was considered when two-sided P < 0.05. R code for the main analyses is included in Supplementary Material for R code (Attachment 1). 

### Ethics

The study was approved by the Ethics Committee of Dongguan Songshan Lake Tungwah Hospital (reference SDHKY-2025-006-01) and is reported following the Strengthening the Reporting of Observational Studies in Epidemiology (STROBE) guidelines (see Supplementary Table S2 in Attachment 1) [23]. 

## Results

### Cohort characteristics

The flowchart of the study is shown in Supplementary Figure S1 (Attachment 1). In all, 278 patients were diagnosed with an MDRPa HAI and 284 with a non-MDRPa HAI between 2018 and 2021. Baseline covariates were considerably imbalanced between the groups (Table 1 (Tab. 1)). The patients with an MDRPa HAI were older, more likely to have been admitted to the ICU with a diagnosis of a respiratory disease, and less likely to have cancer or diabetes than patients with non-MDRPa HAI. Bloodstream and urinary tract infections were more frequent in the MDRPa HAI group. Moreover, the MDRPa HAIs were more prevalent than non-MDRPa HAIs during the post-COVID period. 

The length of hospitalisation for patients with an MDRPa HAI was significantly longer than for patients with a non-MDRPa HAI (median 63 vs. 38.5 days, respectively; *p*<0.001). Length of stay distributions by MDR status and year are shown in Supplementary Figure S2 (Attachment 1). The overall in-hospital mortality was 13.7% (77/562) and was higher in the MDRPa HAI group than the non-MDRPa HAI group (17.3% vs. 10.2%, *P*=0.015).

### Infection incidence rates

**Figure 1:**
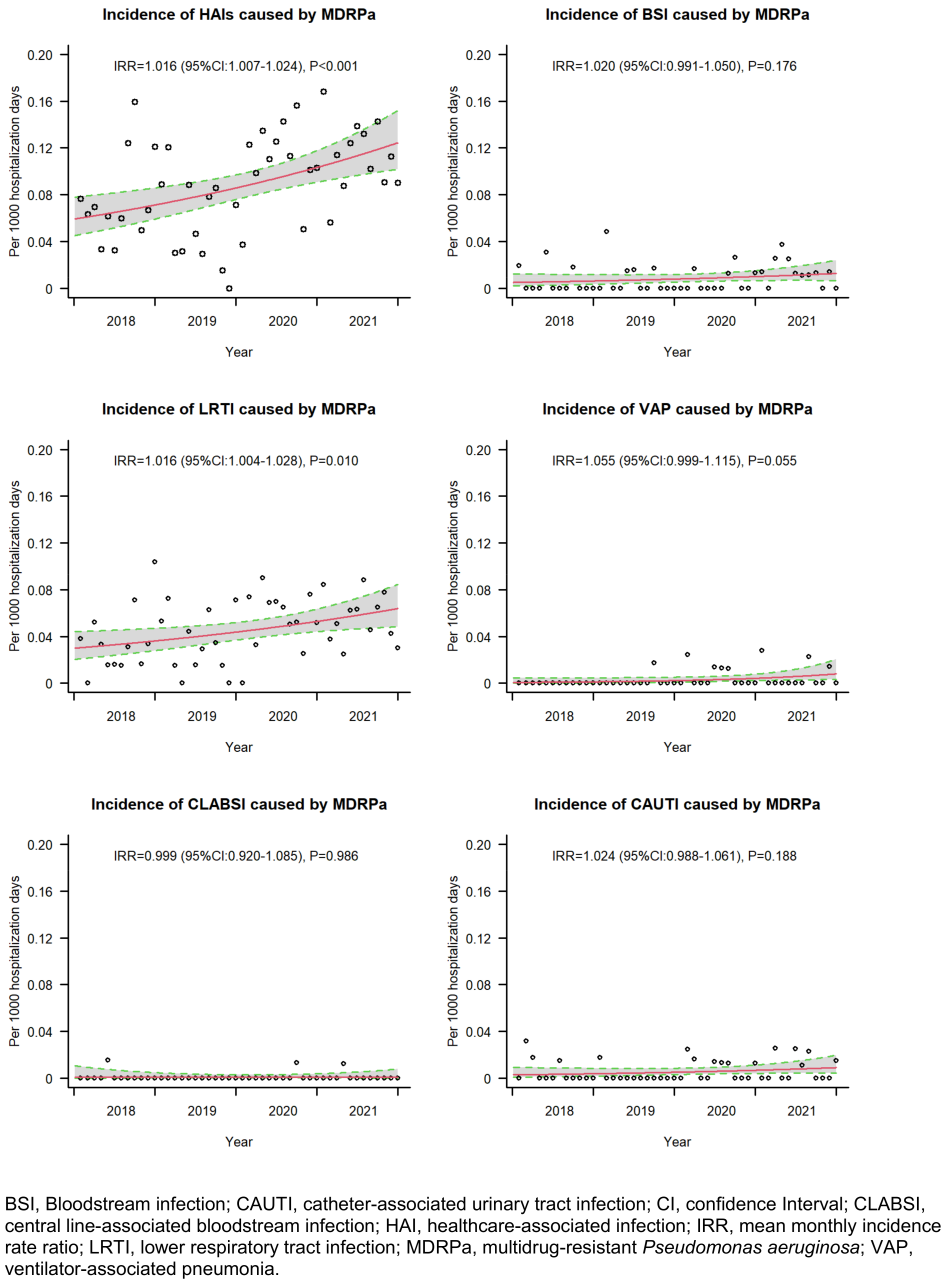
Incidence rate trends of healthcare-associated infection by multidrug-resistant* P. aeruginosa*, 2018–2021

The hospital-wide incidence rate of MDRPa HAIs increased significantly over time, from 0.076 cases per 1,000 hospital-days in 2018 to 0.115 cases per 1,000 hospital-days in 2021, with a mean percentage increase of 1.6% per month (IRR=1.016; 95% CI: 1.007–1.024). The increasing trend was evident for lower respiratory-tract infections and ventilator-associated pneumonias caused by MDRPa (Figure 1 (Fig. 1)). In parallel, non-MDRPa HAI incidence increased over time, mainly due to increasing lower respiratory-tract infections (see Supplementary Figure S3 in Attachment 1). In contrast, the incidence rates of bloodstream infections caused by MDRPa and non-MDRPa remained constant over time.

### Effects on patient outcome

**Figure 2:**
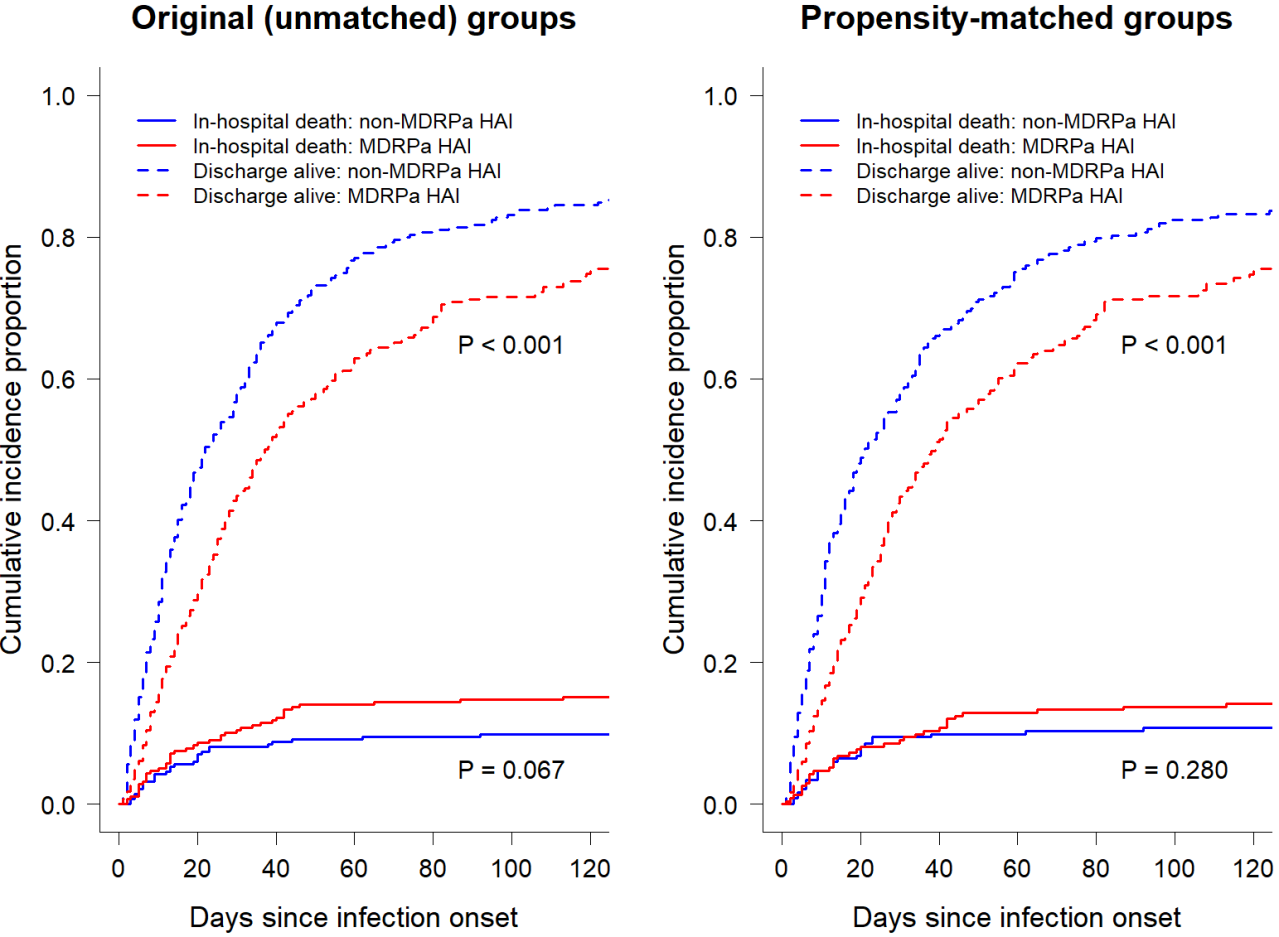
Univariate cumulative function curves showing the probability of each event (in-hospital mortality and discharge alive) over time for MDRPa HAI and non-MDRPa HAI, in both original (unmatched) and propensity-matched groups.

Univariate cumulative function plots (Figure 2 (Fig. 2)) that consider competing risks and the censoring of event times revealed that patients with MDRPa HAI had consistently lower daily probabilities of being discharged alive from the hospital. This implies longer lengths of stay than patients with non-MDRPa HAI, both in the unmatched comparison and in that adjusted for propensity scores. However, less pronounced differences were seen for in-hospital mortality, especially when comparing the propensity-matched groups.

When the Fine-Gray model was applied (Table 2 (Tab. 2)), a slightly higher, albeit statistically non-significant, hazard of 14-day inpatient death (sHR=1.20, 95% CI 0.62–2.35) was found for MDRPa HAI patients in the unmatched analysis, but this was ruled out in the propensity-matched analysis (sHR=1.07, 95% CI 0.52–2.19). Similar results were obtained when the analysis was extended to 30 days from infection onset. However, when the analysis was extended to the entire hospitalisation period, an elevated hazard of in-hospital death was found (sHR=1.37, 95% CI 0.78–2.39 in doubly robust analysis), suggesting that factors other than the infection may become important for the long-term survival of the patients. All the analyses presented in Table 2 (Tab. 2) consistently showed significantly lower subdistribution hazard rates of hospital discharge-alive in the MDRPa HAI group, indicating that these patients experienced longer lengths of hospital stay than non-MDRPa HAI patients. Detailed results from the multivariable Fine-Gray models, including the effects of baseline covariates, are provided in Supplementary Tables S3 and S4 (Attachment 1).

## Discussion

An increase in the prevalence of MDRPa HAIs over the past decade has been repeatedly documented in healthcare settings in China [15], [24], [25]. The present study addresses a critical research gap by providing real-world evidence of the clinical impact of multidrug resistance in HAIs caused by *P. aeruginosa* in a healthcare setting that reflects the typical standard of care in the country. The findings also reflect the current state of antimicrobial stewardship programmes in managing MDRPa HAIs – an area in which limited information is currently available in China.

We observed a significant increase in the frequency of MDRPa HAIs between 2018 and 2021, which is consistent with the increasing burden of hospital-onset MDRPa reported in healthcare settings in the United States during the same period [26]. However, the reasons for this rising incidence in our setting may differ from those in other countries, where increases have been partly attributed to disruptions to healthcare practices and lapses in infection prevention measures associated with COVID-19 [6]. The rise in incidence in the current study is potentially explained by patient characteristics – specifically, an older patient population and a higher prevalence of cardiovascular and cerebrovascular diseases. Our data indicates that patients aged over 65 had significantly lower chances of being discharged alive in the doubly robust analysis (see Supplementary Tables S3 in Attachment 1). This group represents a vulnerable population that is more susceptible to MDRPa HAIs due to prolonged hospitalisation and higher exposure risks. Furthermore, over a third of the patients in our study had cardiovascular and cerebrovascular diseases (Table 1 (Tab. 1)), conditions that are associated with increased 14-day (sHR: 1.65) and 30-day (sHR: 1.18) inpatient mortality, although these increases were not statistically significant. This finding is similar to Denis et al. [27], who reported that ICU patients with cardiovascular diseases and MDRPa infections had an elevated odds ratio (OR: 1.29, 95% CI: 0.84–1.97) for 30-day in-hospital mortality compared to infections caused by susceptible *P. aeruginosa*.

In the doubly robust analysis, we observed that the probability of in-hospital mortality at 14 days was barely elevated (sHR: 1.07) (Table 2 (Tab. 2)), suggesting that MDRPa HAIs may not lead immediately to patient death. Nevertheless, Park et al. [28] reported that adequate empirical antimicrobial therapy within three days significantly reduced 14-day mortality (adjusted OR: 0.23) in patients with *P. aeruginosa* and *Acinetobacter baumannii* bacteraemia in two Korean hospitals, particularly when non-colistin antibiotics were used. Moreover, our study found no significant increase in the hazard for 30-day in-hospital mortality, in contrast to Yuan et al. [29], who reported significantly higher 28-day mortality rates in haematology departments among patients with carbapenem-resistant *P. aeruginosa* BSIs than among those with carbapenem-susceptible infections. This difference likely reflects the immunocompromised status of patients undergoing chemotherapy in Yuan et al.’s study, whereas our patient population had a relatively low proportion of immunocompromised individuals.

Analysing the entire hospitalisation period, we found a non-significantly elevated hazard of overall in-hospital mortality (adjusted sHR: 1.37) but a significantly lower probability of discharge alive (adjusted sHR: 0.66) (Table 2 (Tab. 2)). Similar findings were observed among patients with lower respiratory tract infections caused by MDRPa (see Supplementary Tables S3 in Attachment 1). These results are consistent with von Cube et al. [11], who reported no significant increase in in-hospital mortality hazard (adjusted HR: 1.05) but a significantly lower likelihood of discharge alive (adjusted HR: 0.67) among ICU patients with VAP caused by *P. aeruginosa* than among those with VAP without *P. aeruginosa*. Both our study and von Cube et al. indicate that prolonged hospitalisation potentially elevates the risk of pathogen transmission among patients. This is further reflected in the rising trend in VAP caused by MDRPa that we observed (Figure 1 (Fig. 1)), which underscores the urgent need for MDRPa screening prior to initiating long-term mechanical ventilation and for the reinforcement of stringent infection control measures [30].

The Infectious Diseases Society of America recommends that empirical therapy be guided by clinical judgement and local epidemiological data [31]. Although our analysis – unlike Ohnuma et al. [32] – did not show a significant reduction in in-hospital mortality associated with empirical therapy, we did observe a statistically significant increase in the likelihood of overall discharge alive (sHR: 1.32) (see Supplementary Tables S4 in Attachment 1), similar to findings by Deconinck et al. [33]. These results suggest that appropriate early empirical therapy, followed by prompt de-escalation based on susceptibility test results, may help stabilise patients, prevent clinical deterioration and enhance discharge prospects.

The main strength of the present study is the use of a novel competing risks survival analysis model with propensity-matched and multivariable adjustment, which offers a doubly robust approach to assessing in-hospital mortality [18]. Nonetheless, our study also has limitations. First, certain important baseline covariates, such as Acute Physiology and Chronic Health Evaluation II scores, were unavailable in our dataset, limiting the precision of severity assessments [34]. Second, detailed data on the timing and extent of invasive medical device use before MDRPa HAI diagnosis were lacking, despite evidence suggesting a significant association with increased mortality, particularly interventions like tracheal intubation [35].

In conclusion, this study provides real-world evidence of the clinical impact of MDRPa HAIs in China, highlighting the rising incidence. The findings emphasise the critical need for optimised antimicrobial stewardship programmes to ensure rational antibiotic use. Furthermore, our comprehensive assessment of the MDRPa HAI burden in the context of hospital antimicrobial stewardship offers essential guidance to clinicians to support their evidence-based decision-making in the management of MDRPa HAIs.

## Notes

### Authors’ ORCIDs

- Kritsotakis EI: 0000-0002-9526-3852
- Zhou B: 0000-0001-8853-0724
- Brinks R: 0000-0003-0961-6592
- Wang J: 0000-0001-6284-9702

### Ethical approval 

The study was approved by the Ethics Committee of Dongguan Songshan Lake Tungwah Hospital (reference: SDHKY-2025-006-01). Because the study was based on anonymised routine surveillance data collected as part of healthcare quality improvement programs, the requirement to obtain informed consent was waived. 

### Data protection statement

Due to data protection regulations, patient-level data analysed in this research cannot be shared publicly or with third parties outside the current research group. The datasets can be shared for research purposes upon reasonable request, adhering to the Chinese Clinical Research Ethics Committee standards, and provided that a data transfer agreement from the legal department of the Dongguan Tungwah Hospitals is signed and accepted

### Funding

None. 

### Acknowledgments

The authors gratefully thank the frontline infection control physicians, nurses and colleagues working for the antimicrobial stewardship program for their collaboration and their everlasting. 

### Competing interests

The authors declare that they have no competing interests.

## Supplementary Material

Supplementary materials

## Figures and Tables

**Table 1:**
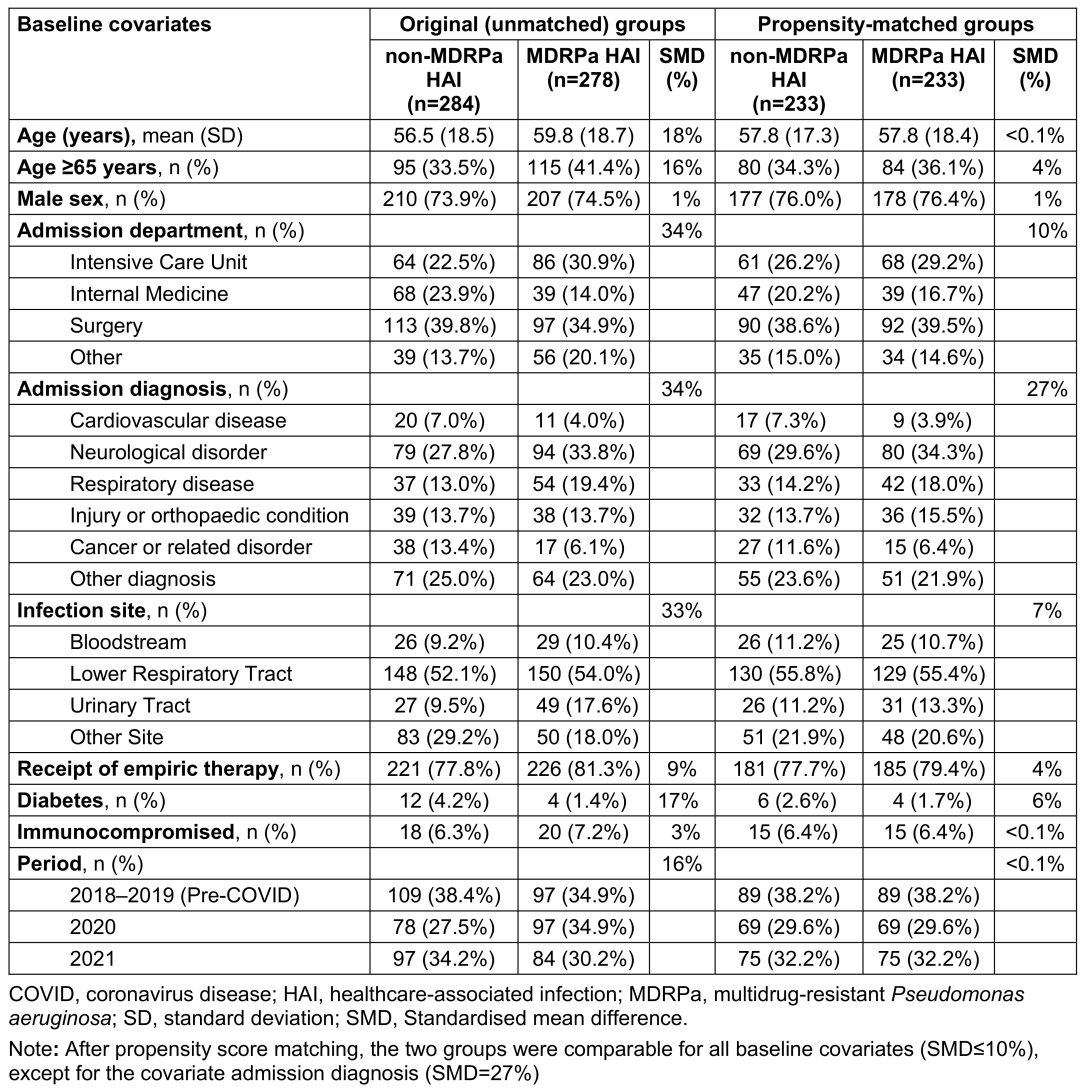
Baseline characteristics of the original and propensity-matched groups of patients infected with *P. aeruginosa* by multidrug resistance status

**Table 2:**
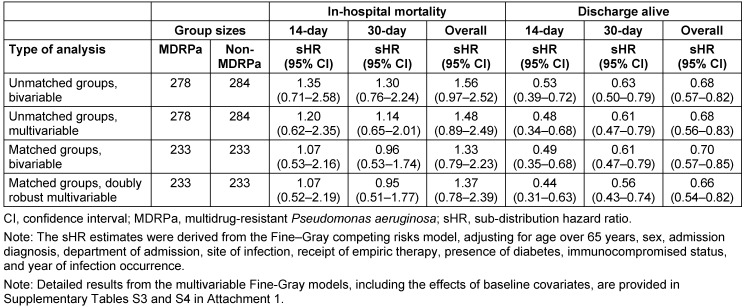
Estimated effects of the multidrug resistance status in *P. aeruginosa* infection on patient outcomes

## References

[1] Raman G, Avendano EE, Chan J, Merchant S, Puzniak L (2018). Risk factors for hospitalized patients with resistant or multidrug-resistant Pseudomonas aeruginosa infections: a systematic review and meta-analysis. Antimicrob Resist Infect Control.

[2] Nelson RE, Hatfield KM, Wolford H, Samore MH, Scott RD, Reddy SC, Olubajo B, Paul P, Jernigan JA, Baggs J (2021). National Estimates of Healthcare Costs Associated With Multidrug-Resistant Bacterial Infections Among Hospitalized Patients in the United States. Clin Infect Dis.

[3] World Health Organization (2024). WHO Bacterial Priority Pathogens List, 2024: Bacterial pathogens of public health importance to guide research, development and strategies to prevent and control antimicrobial resistance.

[4] Tacconelli E, Carrara E, Savoldi A, Harbarth S, Mendelson M, Monnet DL, Pulcini C, Kahlmeter G, Kluytmans J, Carmeli Y, Ouellette M, Outterson K, Patel J, Cavaleri M, Cox EM, Houchens CR, Grayson ML, Hansen P, Singh N, Theuretzbacher U, Magrini N, WHO Pathogens Priority List Working Group (2018). Discovery, research, and development of new antibiotics: the WHO priority list of antibiotic-resistant bacteria and tuberculosis. Lancet Infect Dis.

[5] Schwartz B, Klamer K, Zimmerman J, Kale-Pradhan PB, Bhargava A (2024). Multidrug Resistant Pseudomonas aeruginosa in Clinical Settings: A Review of Resistance Mechanisms and Treatment Strategies. Pathogens.

[6] Ng QX, Ong NY, Lee DYX, Yau CE, Lim YL, Kwa ALH, Tan BH (2023). Trends in Pseudomonas aeruginosa (P. aeruginosa) Bacteremia during the COVID-19 Pandemic: A Systematic Review. Antibiotics (Basel).

[7] Karakonstantis S, Kritsotakis EI, Tziolos RN, Vassilopoulou L, Loukaki M, Kypraiou D, Petrakis EC, Tovil A, Kokkini S, Tryfinopoulou K, Ioannou P, Kondili Ε, Kofteridis DP (2025). Mortality due to carbapenem-resistant Acinetobacter baumannii bacteraemia: a 5-year cohort study in intensive care patients. Clin Microbiol Infect.

[8] Lau B, Cole SR, Gange SJ (2009). Competing Risk Regression Models for Epidemiologic Data. Am J Epidemiol.

[9] Wang J, Zhou M, Hesketh T, Kritsotakis EI (2021). Mortality associated with third generation cephalosporin-resistance in Enterobacteriaceae infections: a multicenter cohort study in Southern China. Expert Rev Anti Infect Ther.

[10] Karakonstantis S, Gikas A, Astrinaki E, Kritsotakis EI (2020). Excess mortality due to pandrug-resistant Acinetobacter baumannii infections in hospitalized patients. J Hosp Infect.

[11] von Cube MK, Timsit JF, Sommer H, Darmon M, Schwebel C, Bailly S, Souweine B, Wolkewitz M (2018). Relative risk and population-attributable fraction of ICU death caused by susceptible and resistant Pseudomonas aeruginosa ventilator-associated pneumonia: a competing risks approach to investigate the OUTCOMEREA database. Intensive Care Med.

[12] Kritsotakis EI, Lagoutari D, Michailellis E, Georgakakis I, Gikas A (2022). Burden of multidrug and extensively drug-resistant ESKAPEE pathogens in a secondary hospital care setting in Greece. Epidemiol Infect.

[13] Magiorakos AP, Srinivasan A, Carey RB, Carmeli Y, Falagas ME, Giske CG, Harbarth S, Hindler JF, Kahlmeter G, Olsson-Liljequist B, Paterson DL, Rice LB, Stelling J, Struelens MJ, Vatopoulos A, Weber JT, Monnet DL (2012). Multidrug-resistant, extensively drug-resistant and pandrug-resistant bacteria: an international expert proposal for interim standard definitions for acquired resistance. Clin Microbiol Infect.

[14] Zhou M, Xu B, Guo Z, Zeng Y, Lei J, Kritsotakis EI, Wang J (2024). Clinical burden of community-associated infections caused by multidrug-resistant Pseudomonas aeruginosa: a propensity-matched longitudinal cohort study in Southern China. GMS Hyg Infect Control.

[15] Wang J, Zhou M, Huang G, Guo Z, Sauser J, Metsini A, Pittet D, Zingg W (2020). Antimicrobial resistance in southern China: results of prospective surveillance in Dongguan city, 2017. J Hosp Infect.

[16] Wang J, Hu J, Harbarth S, Pittet D, Zhou M, Zingg W (2017). Burden of healthcare-associated infections in China: results of the 2015 point prevalence survey in Dong Guan City. J Hosp Infect.

[17] Ministry of Health of the People’s Republic of China (2001). Diagnostic Criteria for Nosocomial Infection (Trial) (In Chinese). Nat Med J China.

[18] Benedetto U, Head SJ, Angelini GD, Blackstone EH (2018). Statistical primer: propensity score matching and its alternatives. Eur J Cardiothorac Surg.

[19] Austin PC (2009). Balance diagnostics for comparing the distribution of baseline covariates between treatment groups in propensity-score matched samples. Stat Med.

[20] Wolkewitz M, Cooper BS, Bonten MJ, Barnett AG, Schumacher M (2014). Interpreting and comparing risks in the presence of competing events. BMJ.

[21] Batomen B, Moore L, Strumpf E, Nandi A (2021). Addressing Competing Risks When Assessing the Impact of Health Services Interventions on Hospital Length of Stay. Epidemiology.

[22] Austin PC, Fine JP (2017). Practical recommendations for reporting Fine-Gray model analyses for competing risk data. Stat Med.

[23] von Elm E, Altman DG, Egger M, Pocock SJ, Gøtzsche PC, Vandenbroucke JP (2007). The Strengthening the Reporting of Observational Studies in Epidemiology (STROBE) statement: guidelines for reporting observational studies. Lancet.

[24] Peng Y, Shi J, Bu T, Li Y, Ye X, Chen X, Yao Z (2015). Alarming and increasing prevalence of multidrug-resistant Pseudomonas aeruginosa among healthcare-associated infections in China: A meta-analysis of cross-sectional studies. J Glob Antimicrob Resist.

[25] Lyu J, Chen H, Bao J, Liu S, Chen Y, Cui X, Guo C, Gu B, Li L (2023). Clinical Distribution and Drug Resistance of Pseudomonas aeruginosa in Guangzhou, China from 2017 to 2021. J Clin Med.

[26] Centers for Disease Control and Prevention (2025). Antimicrobial Resistance Threats in the United States, 2021-2022.

[27] Denis JB, Lehingue S, Pauly V, Cassir N, Gainnier M, Léone M, Daviet F, Coiffard B, Baron S, Guervilly C, Forel JM, Roch A, Papazian L (2019). Multidrug-resistant Pseudomonas aeruginosa and mortality in mechanically ventilated ICU patients. Am J Infect Control.

[28] Park JH, Choi SH, Chung JW (2013). The impact of early adequate antimicrobial therapy on 14-day mortality in patients with monomicrobial Pseudomonas aeruginosa and Acinetobacter baumannii bacteremia. J Infect Chemother.

[29] Yuan F, Li M, Wang X, Fu Y (2024). Risk factors and mortality of carbapenem-resistant Pseudomonas aeruginosa bloodstream infection in haematology department: A 10-year retrospective study. J Glob Antimicrob Resist.

[30] Klompas M, Branson R, Cawcutt K, Crist M, Eichenwald EC, Greene LR, Lee G, Maragakis LL, Powell K, Priebe GP, Speck K, Yokoe DS, Berenholtz SM (2022). Strategies to prevent ventilator-associated pneumonia, ventilator-associated events, and nonventilator hospital-acquired pneumonia in acute-care hospitals: 2022 Update. Infect Control Hosp Epidemiol.

[31] Tamma PD, Heil EL, Justo JA, Mathers AJ, Satlin MJ, Bonomo RA (2024). Infectious Diseases Society of America 2024 Guidance on the Treatment of Antimicrobial-Resistant Gram-Negative Infections. Clin Infect Dis.

[32] Ohnuma T, Chihara S, Costin B, Treggiari MM, Bartz RR, Raghunathan K, Krishnamoorthy V (2023). Association of Appropriate Empirical Antimicrobial Therapy With In-Hospital Mortality in Patients With Bloodstream Infections in the US. JAMA Netw Open.

[33] Deconinck L, Meybeck A, Patoz P, Van Grunderbeeck N, Boussekey N, Chiche A, Delannoy PY, Georges H, Leroy O (2017). Impact of combination therapy and early de-escalation on outcome of ventilator-associated pneumonia caused by Pseudomonas aeruginosa. Infect Dis (Lond).

[34] Wolkewitz M, Cooper BS, Palomar-Martinez M, Alvarez-Lerma F, Olaechea-Astigarraga P, Barnett AG, Harbarth S, Schumacher M (2014). Multilevel competing risk models to evaluate the risk of nosocomial infection. Crit Care.

[35] Sarbazi-Golezari A, Namdar P, Yousefian S, Mirzadeh M, Farnood A, Modirian E (2021). Prognosis of patients with tracheal intubation in the emergency department. Trends Anaesth Crit Care.

