# Supplementary material for: In-hospital mortality from healthcare-associated infection by multidrug-resistant Pseudomonas aeruginosa: a competing risks analysis of a 4-year propensity-matched cohort study in southern China
Source: GMS Hyg Infect Control. 2025 Nov 21;20:Doc68. doi: 10.3205/dgkh000597 (PMC12720264; doi:10.3205/dgkh000597)
Supplement: Supplementary materials [file HIC-20-68-s-001.pdf]

Supplementary Materials

In-hospital mortality from healthcare-associated infection by multidrug-resistant *Pseudomonas aeruginosa*: a competing risks analysis of a 4-year propensity-matched cohort study in southern China

Zhou M, Kritsotakis EI, Xu B, Guo Z, Zeng Y, Zhou B, Brinks R, Wang J

Table of Contents

R code for main analyses....2

Table S1. Multicollinearity analysis 5

Table S2. STROBE Statement—Checklist of items that should be included in reports of cohort studies 6

Figure S1. Flow chart of data collection and patient selection 8

Figure S2. Length of hospitalisation of patients with healthcare-associated infections by *P. aeruginosa* 9

Figure S3. Incidence rate trends for healthcare-associated infections by non-multidrug-resistant *P. aeruginosa* (non-MDRPA), 2018-2021 11

Table S3. Results of multivariable competing risks survival analysis in the matched samples 13

Table S4. Results of multivariable competing risks analysis in the unmatched samples....14

R code for main analyses

```
library("MatchIt")

# Matching based on the estimated propensity scores
formula <- mdr ~ age + sex + admission_department + infection_site + diabetes + immunocompromised + COVID_period
matchit_model <- matchit(formula,
                         data=df,
                         distance='logit',
                         method='nearest',
                         replace=FALSE,
                         caliper=0.2,
                         discard=,
                         ratio=1)

# Check the balance after matching
summary(matchit_model, standardize = TRUE)
```

Competing risks 14-day survival analysis after matching

```
library(survival)

# survival analysis variables
# define time and event variables (from infection onset to death/dicharge)
df2$dthtm= as.numeric(round(df2$discharge_date - df2$infection_date))
df2$dth <- ifelse(df2$alive_mortality == 1, 1, 0) # alive_mortality == 1 = died [dth==1 = died]

# admin right censoring at 14 days after infection
df2$dthtm14 <- df2$dthtm
df2$dthtm14[df2$dthtm14 > 14]  <- 14
df2$dth14 <- ifelse(df2$dthtm<=14 & df2$dth ==1, 2, 1)
df2$dth14[df2$dthtm>14] <- 0

#14d in-hospital death
# bivariable analysis
fgmodel_mtch_univr1_d14 <- FGR(Hist(dthtm14, dth14) ~ mdr , data = df2, cause = 2)        # dth14=2 when inpatient death
print(fgmodel_mtch_univr1_d14)
```

```
# multivariable analysis

fgmodel_mtch_multivar1_d14 <- FGR(Hist(dthtm14, dth14) ~ mdr + infection_site + empiric_therapy
                        + age65 + sex + admission_department + admission_diagnosis + diabetes + immunocompromised
                        + COVID_period , data = df2, cause = 2,maxiter=15)  # dth14=2 when inpatient death

print(fgmodel_mtch_multivar1_d14)
```

#14d discharge alive

```
# bivariable analysis

fgmodel_mtch_univr2_d14 <- FGR(Hist(dthtm14, dth14) ~ mdr , data = df2, cause = 1)      # dth14=1 is discharge alive

print(fgmodel_mtch_univr2_d14)

# multivariable analysis

fgmodel_mtch_multivar2_d14 <- FGR(Hist(dthtm14, dth14) ~ mdr + infection_site + empiric_therapy
                        + age65 + sex + admission_department + admission_diagnosis + diabetes + immunocompromised
                        + COVID_period , data = df2, cause = 1)                              # dth14=1 is discharge alive

print(fgmodel_mtch_multivar2_d14)
```

Table S1. Multicollinearity analysis

Variable	Generalised Variance Inflation Factor	Degrees of Freedom	Adjusted Generalized Variance Inflation Factor
Age ≥65 years	1.160	1	1.077
Sex = Male	1.045	1	1.022
Admission Department	1.950	3	1.118
Admission Diagnosis	2.123	5	1.078
Infection Site	1.220	3	1.034
Receipt of empiric therapy	1.287	1	1.134
Diabetes = Yes	1.056	1	1.028
Immunocompromised = Yes	1.150	1	1.073
COVID Period	1.228	2	1.053

Note: Generalised Variance Inflation Factor (GVIF) analysis for the included variables showed no evidence of multicollinearity. The adjusted GVIF values for all variables were close to 1, indicating low correlation between predictors and suggesting that multicollinearity is not an issue for this set of covariates.

Table S2. STROBE Statement—Checklist of items that should be included in reports of cohort studies

	No	Recommendation	Page(s)
**Title and abstract**	1	(*a*) Indicate the study's design with a commonly used term in the title or the abstract	1
		(*b*) Provide in the abstract an informative and balanced summary of what was done and what was found	3
Introduction			
Background/rationale	2	Explain the scientific background and rationale for the investigation being reported	4-5
Objectives	3	State specific objectives, including any prespecified hypotheses	5
Methods			
Study design	4	Present key elements of study design early in the paper	5
Setting	5	Describe the setting, locations, and relevant dates, including periods of recruitment, exposure, follow-up, and data collection	5
Participants	6	(*a*) Give the eligibility criteria, and the sources and methods of selection of participants. Describe methods of follow-up	5-6
		(*b*) For matched studies, give matching criteria and number of exposed and unexposed	7
Variables	7	Clearly define all outcomes, exposures, predictors, potential confounders, and effect modifiers. Give diagnostic criteria, if applicable	5
Data sources/ measurement	8	For each variable of interest, give sources of data and details of methods of assessment (measurement). Describe comparability of assessment methods if there is more than one group	5-6
Bias	9	Describe any efforts to address potential sources of bias	N/A
Study size	10	Explain how the study size was arrived at	N/A
Quantitative variables	11	Explain how quantitative variables were handled in the analyses. If applicable, describe which groupings were chosen and why	5-6
Statistical methods	12	(*a*) Describe all statistical methods, including those used to control for confounding	6-8
		(*b*) Describe any methods used to examine subgroups and interactions	Subgroup modelling of incidence trends for major types of HAIs-MDRPa, Figure 1 and Figure S3
		(*c*) Explain how missing data were addressed	8
		(*d*) If applicable, explain how loss to follow-up was addressed	N/A
		(<u>*e*</u>) Describe any sensitivity analyses	Table 2
Results			

Participants	13	(a) Report numbers of individuals at each stage of study—e.g., numbers potentially eligible, examined for eligibility, confirmed eligible, included in the study, completing follow-up, and analysed	8, Table 1
		(b) Give reasons for non-participation at each stage	Supplementary Figure S1
		(c) Consider use of a flow diagram	Supplementary Figure S1
Descriptive data	14	(a) Give characteristics of study participants (e.g., demographic, clinical, social) and information on exposures and potential confounders	8
		(b) Indicate number of participants with missing data for each variable of interest	N/A
		(c) Summarise follow-up time (e.g., average and total amount)	N/A
Outcome data	15	Report numbers of outcome events or summary measures over time	8-9
Main results	16	(*a*) Give unadjusted estimates and, if applicable, confounder-adjusted estimates and their precision (e.g., 95% confidence interval). Make clear which confounders were adjusted for and why they were included	Table 2 and Supplementary Tables S3 and S4.
		(*b*) Report category boundaries when continuous variables were categorized	N/A
		(*c*) If relevant, consider translating estimates of relative risk into absolute risk for a meaningful time period	We reported subdistribution hazard ratio
Other analyses	17	Report other analyses done—e.g., analyses of subgroups and interactions, and sensitivity analyses	We did subgroup analysis for incidence rate (Figure 1 and Figure S3).
Discussion			
Key results	18	Summarise key results with reference to study objectives	10-11
Limitations	19	Discuss limitations of the study, taking into account sources of potential bias or imprecision. Discuss both direction and magnitude of any potential bias	12
Interpretation	20	Give a cautious overall interpretation of results considering objectives, limitations, multiplicity of analyses, results from similar studies, and other relevant evidence	10-11
Generalisability	21	Discuss the generalisability (external validity) of the study results	N/A
Other information			
Funding	22	Give the source of funding and the role of the funders for the present study and, if applicable, for the original study on which the present article is based	13

Figure S1. Flow chart of data collection and patient selection

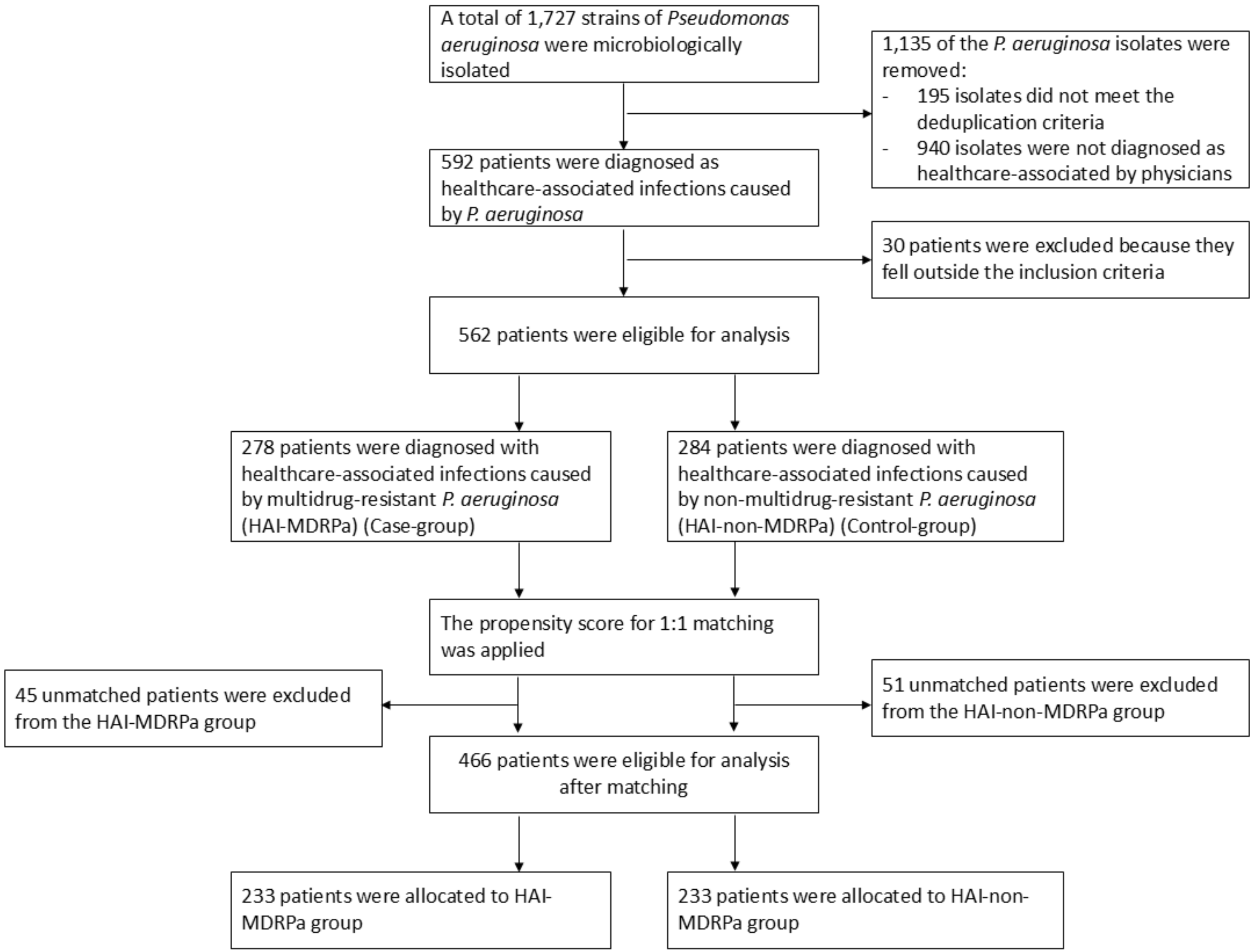

Figure S2. Length of hospitalisation of patients with healthcare-associated infections by *P. aeruginosa*

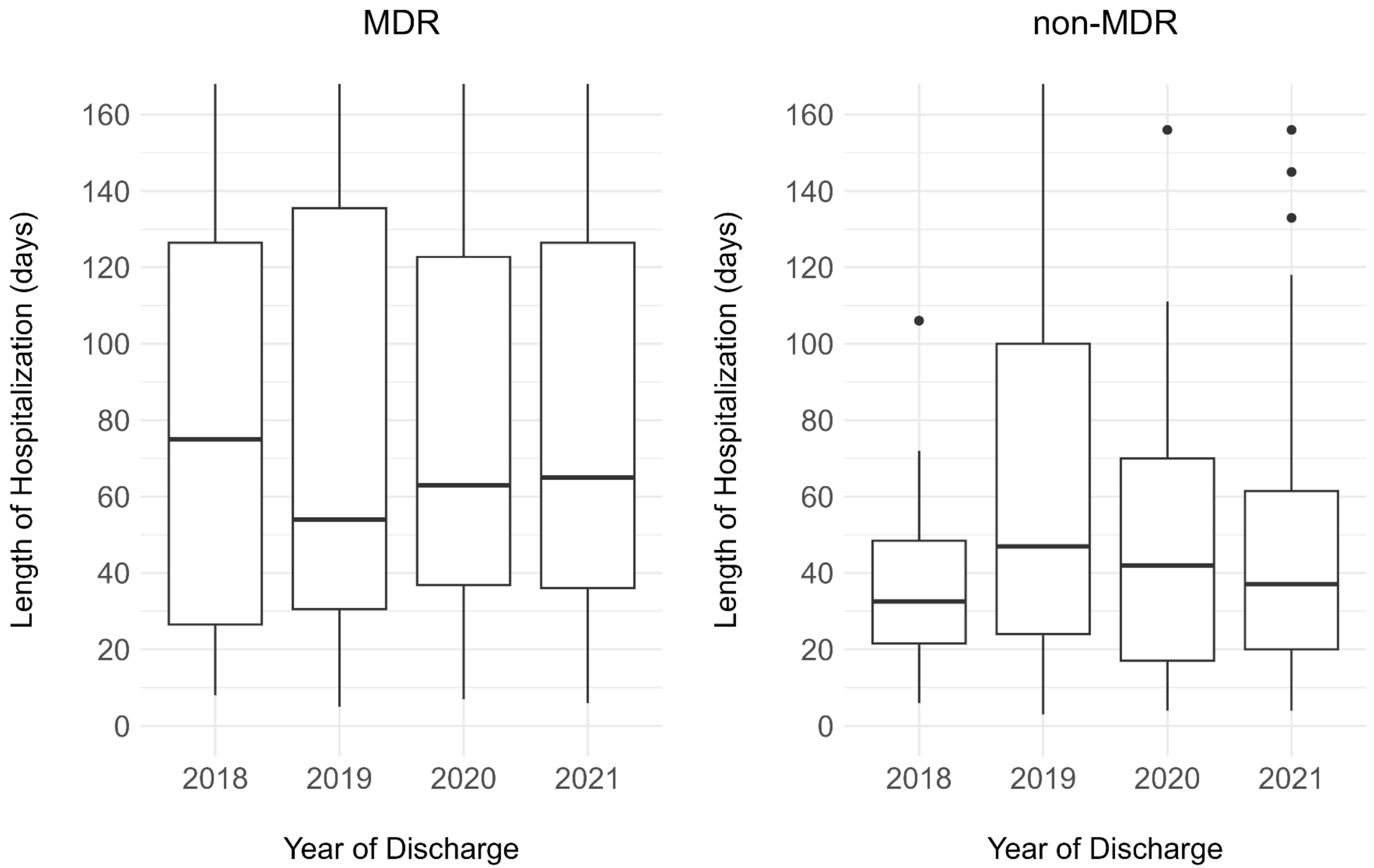

Attachment 1 to: *Zhou M, Kritsotakis EI, Xu B, Guo Z, Zeng Y, Zhou B, Brinks R, Wang J. In-hospital mortality from healthcare-associated infection by multidrug-resistant Pseudomonas aeruginosa: a competing risks analysis of a 4-year propensity-matched cohort study in southern China. GMS Hyg Infect Control. 2025;20:Doc68. DOI: 10.3205/ddgkh000597*

MDR, multidrug resistance

Figure S3. Incidence rate trends for healthcare-associated infections by non-multidrug-resistant *P. aeruginosa* (non-MDRPA), 2018-2021

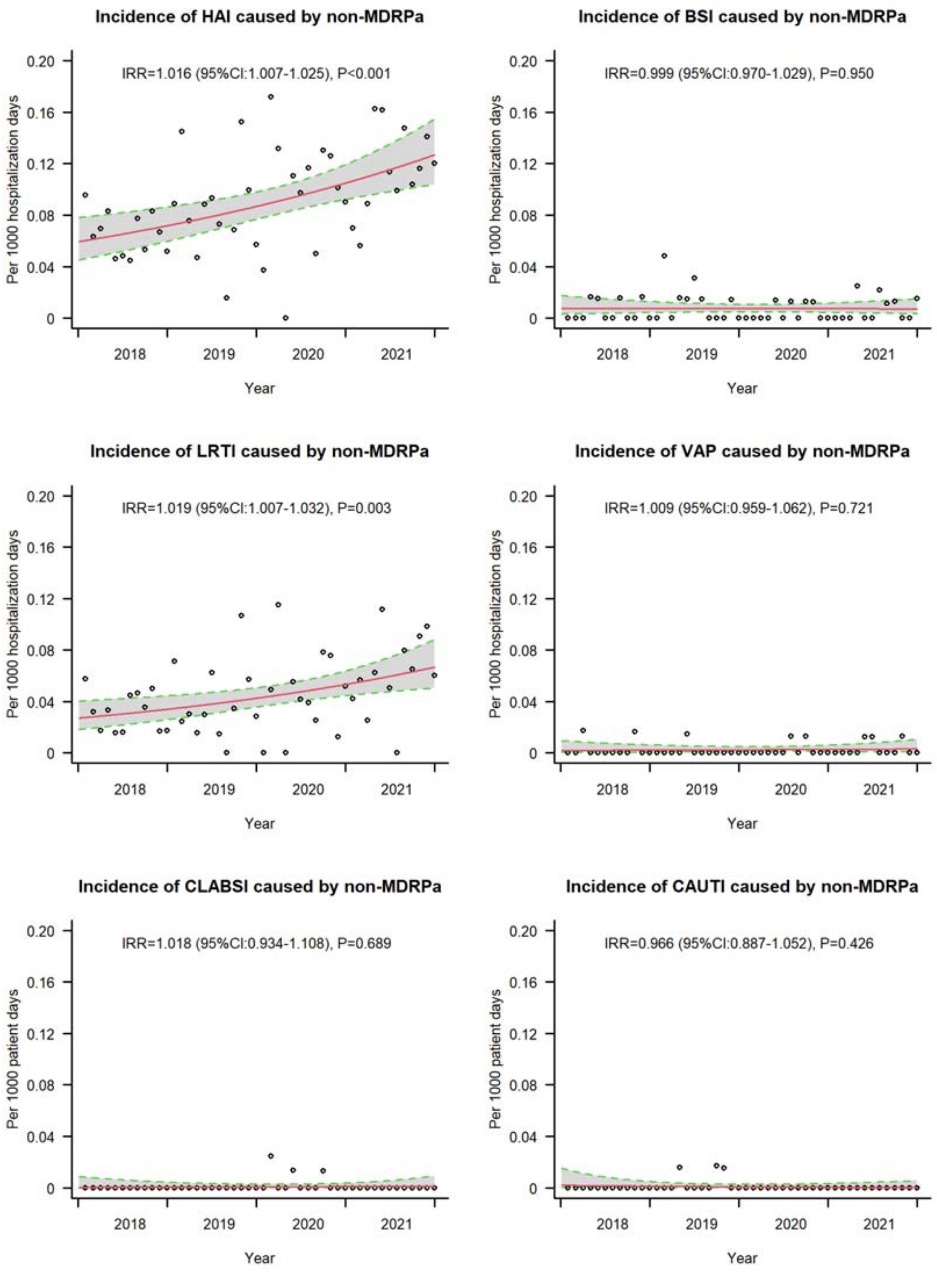

BSI, Bloodstream infection; CAUTI, catheter-associated urinary tract infection; CI, confidence Interval; CLABSI, central line-associated bloodstream infection; HAI, healthcare-associated

Attachment 1 to: *Zhou M, Kritsotakis EI, Xu B, Guo Z, Zeng Y, Zhou B, Brinks R, Wang J. In-hospital mortality from healthcare-associated infection by multidrug-resistant Pseudomonas aeruginosa: a competing risks analysis of a 4-year propensity-matched cohort study in southern China. GMS Hyg Infect Control. 2025;20:Doc68. DOI: 10.3205/dgkh000597*

infection; IRR, mean monthly incidence rate ratio; LRTI, lower respiratory tract infection; MDRPa, multidrug-resistant *Pseudomonas aeruginosa*; VAP, ventilator-associated pneumonia.

Table S3. Results of multivariable competing risks survival analysis in the matched samples

	14 Days				30 Days				Overall			
	In-hospital death		Discharge alive		In-hospital death		Discharge alive		In-hospital death		Discharge alive	
**Variable**	**sHR (95%CI)**	**P**	**sHR (95%CI)**	**P**	**sHR (95%CI)**	**P**	**sHR (95%CI)**	**P**	**sHR (95%CI)**	**P**	**sHR (95%CI)**	**P**
Multidrug-resistant	1.07 (0.52 - 2.19)	0.850	0.44 (0.31 - 0.63)	<0.001	0.95 (0.51 - 1.77)	0.860	0.56 (0.43 - 0.74)	<0.001	1.37 (0.78 - 2.39)	0.280	0.66 (0.54 - 0.82)	<0.001
Age > 65 years	1.34 (0.67 - 2.72)	0.410	0.55 (0.38 - 0.82)	0.003	1.39 (0.74 - 2.60)	0.300	0.68 (0.51 - 0.91)	0.010	1.64 (0.94 - 2.88)	0.082	0.70 (0.55 - 0.89)	0.004
Male Sex	1.21 (0.49 - 3.00)	0.680	0.87 (0.58 - 1.31)	0.510	1.35 (0.60 - 3.00)	0.470	0.90 (0.66 - 1.22)	0.480	1.19 (0.63 - 2.22)	0.600	0.89 (0.69 - 1.14)	0.350
Admission Department												
Internal Medicine	0.04 (0.00 - 0.35)	0.004	1.01 (0.58 - 1.75)	0.980	0.05 (0.01 - 0.20)	<0.001	1.21 (0.81 - 1.82)	0.350	0.11 (0.04 - 0.30)	<0.001	1.93 (1.30 - 2.87)	0.001
Surgery	0.09 (0.03 - 0.31)	<0.001	0.75 (0.45 - 1.26)	0.270	0.08 (0.03 - 0.23)	<0.001	0.76 (0.50 - 1.16)	0.210	0.11 (0.05 - 0.25)	<0.001	1.51 (1.04 - 2.19)	0.030
Other	0.00 (0.00 - 0.00)	<0.001	0.61 (0.34 - 1.11)	0.110	0.00 (0.00 - 0.00)	<0.001	0.69 (0.44 - 1.07)	0.098	0.06 (0.01 - 0.28)	<0.001	1.47 (1.02 - 2.10)	0.036
Admission Diagnosis												
Neurological disorder	1.65 (0.32 - 8.41)	0.550	0.63 (0.28 - 1.42)	0.270	1.18 (0.34 - 4.08)	0.790	0.64 (0.35 - 1.17)	0.150	1.07 (0.36 - 3.16)	0.910	0.76 (0.44 - 1.31)	0.320
Respiratory disease	0.84 (0.18 - 4.01)	0.830	1.72 (0.78 - 3.78)	0.180	0.55 (0.16 - 1.85)	0.330	1.33 (0.72 - 2.48)	0.360	0.54 (0.19 - 1.58)	0.260	1.29 (0.71 - 2.34)	0.400
Injury or orthopaedic condition	0.39 (0.03 - 5.74)	0.490	0.65 (0.28 - 1.53)	0.320	0.48 (0.07 - 3.37)	0.460	0.70 (0.36 - 1.35)	0.290	0.26 (0.04 - 1.68)	0.160	0.91 (0.52 - 1.60)	0.750
Cancer or related disorder	2.44 (0.38 - 15.76)	0.350	0.71 (0.29 - 1.73)	0.450	1.59 (0.36 - 7.06)	0.540	0.68 (0.34 - 1.37)	0.280	1.43 (0.38 - 5.39)	0.590	0.65 (0.34 - 1.23)	0.190
Other diagnosis	1.71 (0.32 - 9.19)	0.530	1.23 (0.58 - 2.60)	0.590	0.97 (0.26 - 3.66)	0.970	1.30 (0.74 - 2.29)	0.360	1.14 (0.38 - 3.47)	0.810	1.24 (0.73 - 2.12)	0.420
Infection Site												
Lower Respiratory Tract	1.09 (0.28 - 4.23)	0.900	0.55 (0.35 - 0.86)	0.008	0.99 (0.36 - 2.72)	0.990	0.61 (0.43 - 0.88)	0.008	1.08 (0.42 - 2.82)	0.870	0.69 (0.51 - 0.95)	0.021
Urinary Tract	1.80 (0.37 - 8.74)	0.470	1.01 (0.55 - 1.85)	0.990	1.00 (0.25 - 4.02)	1.000	0.73 (0.43 - 1.26)	0.260	1.59 (0.50 - 5.13)	0.430	0.64 (0.41 - 0.99)	0.043
Other Site	2.35 (0.55 - 10.07)	0.250	0.97 (0.60 - 1.58)	0.900	1.86 (0.58 - 5.95)	0.300	0.90 (0.60 - 1.37)	0.640	1.96 (0.67 - 5.77)	0.220	0.79 (0.55 - 1.14)	0.210
Receipt of empiric therapy	0.65 (0.23 - 1.84)	0.420	1.29 (0.73 - 2.25)	0.380	0.79 (0.33 - 1.90)	0.600	1.11 (0.74 - 1.67)	0.610	0.63 (0.31 - 1.28)	0.200	1.22 (0.93 - 1.61)	0.160
Diabetes	1.46 (0.34 - 6.26)	0.610	0.81 (0.22 - 2.97)	0.750	1.97 (0.67 - 5.79)	0.220	1.06 (0.45 - 2.49)	0.900	2.11 (0.66 - 6.73)	0.210	0.93 (0.44 - 1.94)	0.840
Immunocompromised	1.97 (0.45 - 8.67)	0.370	1.23 (0.59 - 2.57)	0.580	2.98 (0.76 - 11.76)	0.120	1.14 (0.67 - 1.95)	0.640	1.32 (0.34 - 5.09)	0.690	1.16 (0.78 - 1.73)	0.450
Year												
2020	0.50 (0.20 - 1.22)	0.130	0.89 (0.56 - 1.39)	0.600	0.54 (0.24 - 1.22)	0.140	0.95 (0.67 - 1.36)	0.790	0.60 (0.29 - 1.25)	0.170	1.24 (0.95 - 1.61)	0.120
2021	0.46 (0.18 - 1.15)	0.098	1.27 (0.85 - 1.89)	0.250	0.44 (0.20 - 0.95)	0.036	1.43 (1.04 - 1.97)	0.030	0.46 (0.23 - 0.92)	0.028	1.64 (1.25 - 2.15)	<0.001

Note: The reference group is the intensive care unit for the admission department, cardiovascular disease for the admission diagnosis, bloodstream infection for the infection site, and 2018-2019 for the year

sHR: subdistribution hazard ratio from the Fine-Gray model; 95% CI: 95% confidence interval.

Table S4. Results of multivariable competing risks analysis in the unmatched samples

	14 Days				30 Days				Overall			
	In-hospital death		Discharge alive		In-hospital death		Discharge alive		In-hospital death		Discharge alive	
**Variable**	**sHR (95%CI)**	**P**	**sHR (95%CI)**	**P**	**sHR (95%CI)**	**P**	**sHR (95%CI)**	**P**	**sHR (95%CI)**	**P**	**sHR (95%CI)**	**P**
Multidrug-resistant	1.20 (0.62 - 2.35)	0.590	0.48 (0.34 - 0.68)	<0.001	1.14 (0.65 - 2.01)	0.640	0.61 (0.47 - 0.79)	<0.001	1.48 (0.89 - 2.49)	0.130	0.68 (0.56 - 0.83)	<0.001
Age > 65 years	1.14 (0.60 - 2.19)	0.690	0.68 (0.48 - 0.97)	0.033	1.33 (0.75 - 2.35)	0.330	0.80 (0.61 - 1.04)	0.100	1.58 (0.94 - 2.66)	0.087	0.75 (0.61 - 0.94)	0.011
Male Sex	1.50 (0.64 - 3.53)	0.350	0.80 (0.56 - 1.13)	0.210	1.78 (0.84 - 3.78)	0.140	0.80 (0.61 - 1.04)	0.096	1.53 (0.85 - 2.76)	0.160	0.83 (0.66 - 1.03)	0.097
Admission Department												
Internal Medicine	0.04 (0.01 - 0.24)	<0.001	0.95 (0.58 - 1.57)	0.850	0.05 (0.01 - 0.18)	<0.001	1.03 (0.70 - 1.50)	0.890	0.14 (0.06 - 0.34)	<0.001	1.75 (1.22 - 2.53)	0.002
Surgery	0.07 (0.02 - 0.23)	<0.001	0.76 (0.47 - 1.23)	0.260	0.07 (0.02 - 0.19)	<0.001	0.83 (0.57 - 1.21)	0.330	0.10 (0.04 - 0.22)	<0.001	1.51 (1.08 - 2.12)	0.017
Other	0.00 (0.00 - 0.00)	<0.001	0.69 (0.41 - 1.14)	0.150	0.02 (0.00 - 0.20)	<0.001	0.78 (0.53 - 1.15)	0.210	0.06 (0.02 - 0.21)	<0.001	1.58 (1.14 - 2.19)	0.007
Admission Diagnosis												
Neurological disorder	1.00 (0.25 - 3.95)	1.000	0.66 (0.31 - 1.39)	0.270	0.85 (0.28 - 2.58)	0.780	0.70 (0.39 - 1.24)	0.220	0.68 (0.27 - 1.74)	0.420	0.86 (0.52 - 1.41)	0.540
Respiratory disease	0.65 (0.18 - 2.34)	0.510	1.78 (0.85 - 3.75)	0.130	0.58 (0.20 - 1.69)	0.320	1.39 (0.77 - 2.53)	0.270	0.49 (0.20 - 1.21)	0.120	1.30 (0.74 - 2.28)	0.360
Injury or orthopaedic condition	0.25 (0.02 - 3.17)	0.290	0.62 (0.27 - 1.44)	0.270	0.34 (0.05 - 2.15)	0.250	0.74 (0.39 - 1.39)	0.350	0.17 (0.03 - 1.02)	0.053	0.96 (0.57 - 1.63)	0.890
Cancer or related disorder	1.78 (0.39 - 8.12)	0.450	0.70 (0.31 - 1.61)	0.410	1.34 (0.37 - 4.90)	0.660	0.85 (0.45 - 1.60)	0.610	0.91 (0.29 - 2.91)	0.880	0.81 (0.47 - 1.42)	0.470
Other diagnosis	1.06 (0.28 - 4.01)	0.930	1.15 (0.57 - 2.35)	0.690	0.79 (0.26 - 2.46)	0.690	1.35 (0.79 - 2.32)	0.280	0.79 (0.31 - 2.00)	0.620	1.32 (0.81 - 2.17)	0.270
Infection Site												
Lower Respiratory Tract	0.81 (0.29 - 2.26)	0.690	0.57 (0.37 - 0.87)	0.010	0.79 (0.34 - 1.82)	0.580	0.66 (0.46 - 0.94)	0.020	0.89 (0.40 - 1.96)	0.760	0.74 (0.54 - 1.00)	0.053
Urinary Tract	1.06 (0.29 - 3.90)	0.930	1.01 (0.58 - 1.77)	0.970	0.80 (0.26 - 2.51)	0.700	0.79 (0.48 - 1.29)	0.350	1.25 (0.48 - 3.26)	0.650	0.72 (0.48 - 1.07)	0.110
Other Site	1.56 (0.49 - 4.98)	0.460	0.80 (0.50 - 1.29)	0.370	1.17 (0.43 - 3.19)	0.760	0.85 (0.57 - 1.27)	0.440	1.36 (0.55 - 3.40)	0.510	0.84 (0.60 - 1.20)	0.340
Receipt of empiric therapy	0.59 (0.24 - 1.45)	0.250	1.35 (0.81 - 2.25)	0.250	0.72 (0.33 - 1.55)	0.400	1.27 (0.88 - 1.84)	0.210	0.59 (0.30 - 1.14)	0.120	1.32 (1.03 - 1.68)	0.029
Diabetes	0.78 (0.18 - 3.35)	0.740	0.45 (0.13 - 1.51)	0.200	1.19 (0.40 - 3.51)	0.760	0.72 (0.34 - 1.50)	0.370	1.76 (0.61 - 5.02)	0.290	0.72 (0.39 - 1.34)	0.300
Immunocompromised	5.16 (1.74 - 15.27)	0.003	1.06 (0.55 - 2.04)	0.850	4.46 (1.59 - 12.5)	0.004	0.96 (0.58 - 1.60)	0.880	2.20 (0.78 - 6.22)	0.140	1.00 (0.70 - 1.44)	1.000
Year												
2020	0.52 (0.24 - 1.12)	0.094	0.94 (0.62 - 1.44)	0.790	0.63 (0.33 - 1.21)	0.170	0.97 (0.69 - 1.35)	0.850	0.59 (0.33 - 1.08)	0.086	1.25 (0.97 - 1.60)	0.080
2021	0.36 (0.15 - 0.86)	0.022	1.25 (0.86 - 1.81)	0.240	0.40 (0.19 - 0.83)	0.014	1.43 (1.07 - 1.92)	0.015	0.38 (0.20 - 0.74)	0.004	1.62 (1.27 - 2.07)	<0.001

Note: The reference group is the intensive care unit for the admission department, cardiovascular disease for the admission diagnosis, bloodstream infection for the infection site, and 2018-2019 for the year

sHR: subdistribution hazard ratio from the Fine-Gray model; 95% CI: 95% confidence interval.
